# Effects of Combined Aerobic and Resistance Exercise on Exercise Capacity, Muscle Strength and Quality of Life in HIV-Infected Patients: A Systematic Review and Meta-Analysis

**DOI:** 10.1371/journal.pone.0138066

**Published:** 2015-09-17

**Authors:** Mansueto Gomes Neto, Cristiano Sena Conceição, Vitor Oliveira Carvalho, Carlos Brites

**Affiliations:** 1 Departamento de Biofunçāo, Curso de Fisioterapia da Universidade Federal da Bahia, UFBA, Salvador, BA, Brazil; 2 Programa de Pós Graduação em Medicina e Saúde, UFBA, Salvador, BA, Brazil; 3 The GREAT Group (GRupo de Estudos em ATividade física), Universidade Federal de Sergipe, Aracaju, SE, Brazil; 4 Departamento de Fisioterapia da Universidade Federal de Sergipe, UFS, Aracaju, SE, Brazil; University of Rome Foro Italico, ITALY

## Abstract

**Background:**

Many HIV-infected patients demonstrate disability and lower aerobic capacity. The inclusion of resistance training combined with aerobic exercise in a single program is known as combined aerobic and resistance exercise (CARE) and seems to be an effective strategy to improve muscle weakness, as well as aerobic capacity in HIV-infected patients. We performed a meta-analysis to investigate the effects of CARE in HIV-infected patients.

**Methods:**

We searched MEDLINE, Cochrane Controlled Trials Register, EMBASE, CINAHL (from the earliest date available to august 2014) for controlled trials that evaluated the effects of CARE in HIV-infected patients. Weighted mean differences (WMD) and 95% confidence intervals (CIs) were calculated, and heterogeneity was assessed using the I^2^ test.

**Results:**

Seven studies met the study criteria. CARE resulted in improvement in Peak VO_2_ WMD (4.48 mL·kg^-1^·min^-1^ 95% CI: 2.95 to 6.0), muscle strength of the knee extensors WMD (25.06 Kg 95% CI: 10.46 to 39.66) and elbow flexors WMD (4.44 Kg 95% CI: 1.22 to 7.67) compared with no exercise group. The meta-analyses also showed significant improvement in Health status, Energy/Vitality and physical function domains of quality of life for participants in the CARE group compared with no exercise group. A nonsignificant improvement in social function domain of quality of life was found for participants in the CARE group compared with no exercise group.

**Conclusions:**

Combined aerobic and resistance exercise may improve peak VO_2_, muscle strength and health status, energy and physical function domains of quality of life and should be considered as a component of care of HIV-infected individuals.

## Background

Treatment of HIV disease has undergone substantial advances. Currently, HIV-infection is considered a chronic condition that has been related to disability, decreased exercise capacity and impairment in daily activities [1,2]. Impaired physical functioning is attributed to a combination of several factors, such as structural and inflammatory muscle abnormalities [3], physiological deconditioning [4] and cardiopulmonary dysfunction [5,6].

Exercise training has been considered important for health promotion and rehabilitation of patients with HIV/AIDS. The available evidences suggest that exercise training increases aerobic capacity, muscle function, functional ability and quality of life in HIV-infected patients [7,8]. Although both resistance [9] and aerobic [10] exercise training improve several health outcomes in HIV-infected patients, these two modalities applied as a combined program may be more effective in optimizing functional status than programs involving only one component [11]. A better understanding of the effects of exercise will enable people with HIV and their health care providers to practice effective and appropriate exercise prescription.

Recently we have published a systematic review evaluating the effects of combined aerobic and resistance exercise (CARE) on patients with HIV, however it also included studies that compared exercise to other therapeutic interventions. In addition, meta-analysis was not performed. Since we published our previous systematic review [11,12], new randomized controlled trials (RCTs) have been released. The Cochrane Collaboration recommends that systematic reviews are updated biannually [13]. Moreover, as far as we know, there is no published meta-analysis on the effects of CARE in HIV-infected patients. It is known that meta-analysis technique minimizes subjectivity by standardizing treatment effects of relevant studies into effect sizes (ESs), pooling, and analyzing data to draw conclusions. The aim of this systematic review with meta-analysis was to analyze the published RCTs that investigated the effects of CARE on peak oxygen consumption, muscle performance, and quality of life in HIV-infected patients.

## Methods

This meta-analysis was completed in accordance with Preferred Reporting Items for Systematic Reviews and Meta-Analyses (PRISMA) guidelines [14].

### Eligibility Criteria

This systematic review included RCTs that studied the effects of CARE in HIV-infected patients. To be eligible, the trial should have HIV-infected patients assigned to a group of combined aerobic and resistance exercise. CARE was defined as the application of resistance and aerobic exercise in the same training session [11], performed at least two times per week for at least four weeks. Studies that enrolled patients with cardiac or respiratory diseases were excluded.

The main outcomes of interest were peak oxygen consumption (peak VO_2_, mL/Kg/min), muscle performance, and quality of life.

### Search Methods for Identification of Studies

We searched for references on MEDLINE, PEDro, EMBASE, SciELO, Cumulative Index to Nursing and Allied Health (CINAHL) and the Cochrane Library up to august 2014 without language restrictions. A standard protocol for this search was developed and whenever possible, controlled vocabulary (Mesh term for MEDLINE and Cochrane and EMTREE for EMBASE) were used. Key words and their synonymous were used to sensitize the search.

The strategy developed by Higgins and Green [13] was used for the identification of RCTs in PUBMED/MEDLINE. To identify the RCTs in other data base we adopted a search strategy using similar terms. For the preparation of the search strategy, were used three groups of keywords: study design, participants, and interventions. The search strategy for MEDLINE via PUBMED is presented in Table 1.

**Table 1:** Search strategy MEDLINE via PUBMED.

1. Randomized Controlled Trials
2. Random allocation
3. Controlled Clinical Trials
4. Control groups
5. Clinical trials/ or clinical trials, phase i/ or clinical trials, phase ii/ or clinical trials, phase iii/ or clinical trials, phase iv
6. Clinical Trials Data Monitoring Committees
7. Double-blind method
8. Single-blind method
9. Placebos
10. Placebo effect
11. Cross-over studies
12. Multicenter Studies
13. 1 OR 2 OR 3 OR 4 OR 5 OR 6 OR 7 OR 8 OR 9 OR 10 OR 11 OR 12
14. Human immunodeficiency virus
15. Acquired immunodeficiency syndrome
16. Aids
17. Hiv
18. Retroviruses
19. Hiv infections
20. 14 OR 15 OR 16 OR 17 OR 18 OR 19
21. Exercise
22. physical fitness
23. training
24. Resistance
25. Aerobic
26. 21 OR 22 OR 23 OR 24 OR 25
27. 13 AND 20 AND 26

We checked the references used in articles included in this meta-analysis to identify other potentially eligible studies. For ongoing studies, confirmation of any data or getting additional information, authors were contacted by e-mail.

### Data Collection and Analysis

The previously described search strategy was used to obtain titles and abstracts of studies that might be relevant for this review. Each identified abstract was independently evaluated by two authors. If at least one of the authors considered one reference eligible, the full text was obtained for complete assessment. Two reviewers independently assessed the full text of selected articles to verify if they met the criteria for inclusion or exclusion. In case of any disagreement, authors discussed the reasons for their decisions and a consensual decision was made.

Two authors independently extracted data from the published reports using standard data extraction forms adapted from Higgins and Green [13]. Aspects of the study population, intervention performed, follow-up period and rates of missing data, outcome measures, and results were reviewed. Disagreements were resolved by one of the authors. Any further information required from the original author was requested by e-mail.

### Risk of Bias of Included Studies

The risk of bias in included studies was assessed independently by two reviewers using The Cochrane Collaboration’s ‘Risk of bias’ tool [13]. The following criteria were considered: Random sequence generation, allocation concealment, blinding of participants and personnel, blinding of outcome assessment, incomplete outcome data, selective reporting, intention-to-treat analysis and completeness of follow-up.

### Quality of Meta-Analysis Evidence

The quality of studies included in this systematic review was scored by two researchers using the PEDro scale which is based on important criteria, such as concealed allocation, intention-to-treat analysis, and the adequacy of follow-up. These characteristics make the PEDro scale a useful tool for assessing the quality of physiotherapy and rehabilitation trials [15].

The PEDro scale consisted of 11 items and is based on a Delphi list [16]. One item on the PEDro scale (eligibility criteria) is related to external validity and it generally is not used to calculate the score, leaving a score range of 0 to 10 [17]. Any disagreements were resolved by a third rater.

### Statistical Assessment

Pooled-effect estimates were obtained by comparing the least square mean percentage change from baseline to study end for each group, and were expressed as the weighted mean difference between groups. Calculations were done using a random-effects model. When the SD of change was not available, the SD of the baseline measure was used for the meta-analysis. We compared CARE versus control group (no exercise). An α value of 0.05 was considered significant. Statistical heterogeneity of the treatment effect among studies was assessed using Cochran's Q-test and the inconsistency *I* ^2^ test, in which values between 25 and 50% were considered indicative of moderate heterogeneity, and values greater than 50% were considered indicative of high heterogeneity [18]. All analyses were conducted using Review Manager Version 5.0 (Cochrane Collaboration) [19].

## Results

### Description of Selected Studies

**Fig 1:**
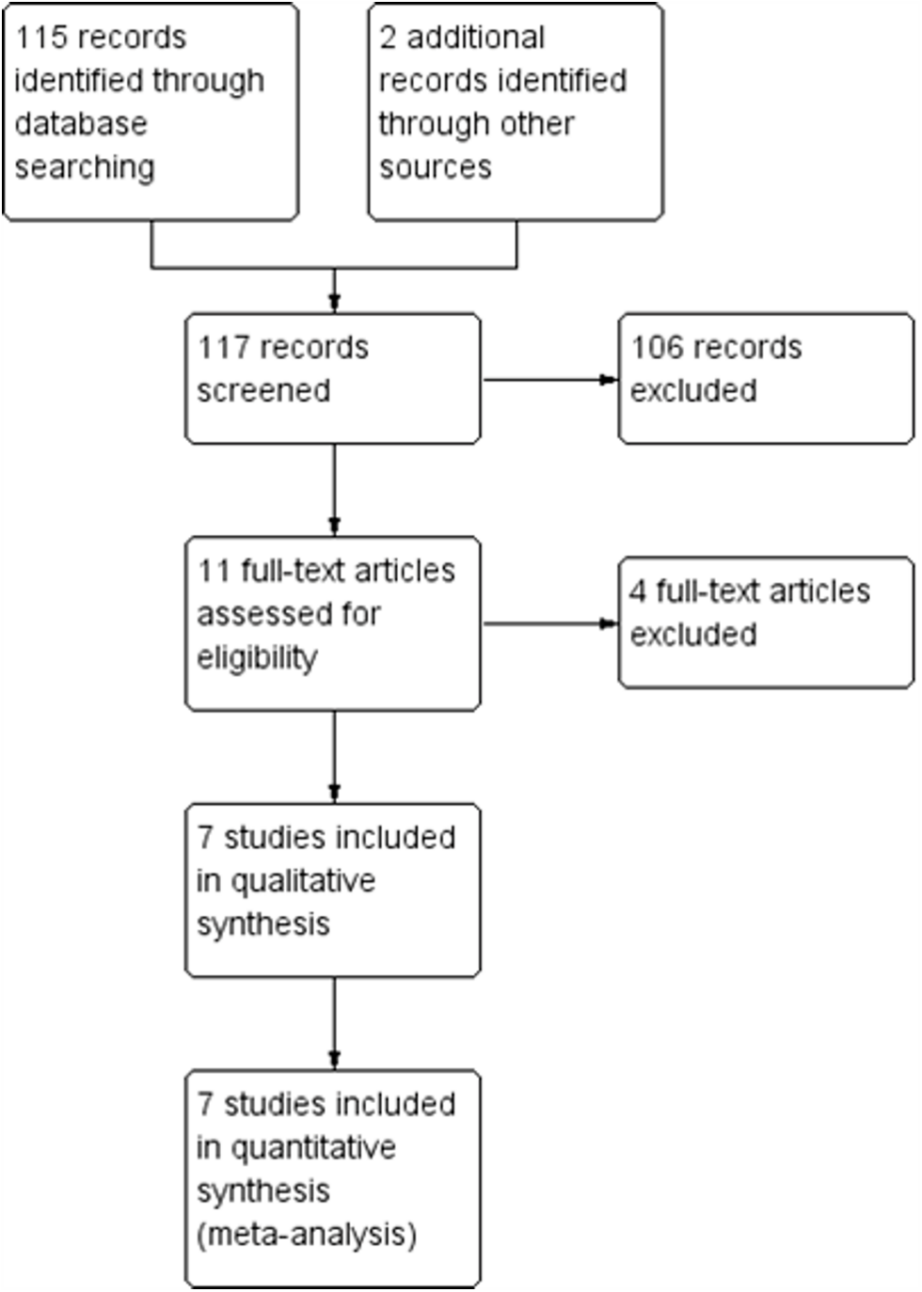
Search and selection of studies for systematic review according PRISMA.

The initial search led to the identification of 117 abstracts, from which 11 studies were considered as potentially relevant and were retrieved for detailed analysis. After a complete reading of 11 articles, 4 were excluded. Two studies were excluded because they linked exercise to other therapies, such as the use of metformin [20] and testosterone [21]. One study was excluded due to inclusion of HIV/HCV co-infected patients [22]. Another study was also excluded from our review because investigated patients with and without HIV [23]. Finally, seven papers [24–30] met the eligibility criteria. Fig 1. shows the PRISMA flow diagram of studies in this review. Table 2 presents results of individual assessment by PEDro scale.

**Table 2:** Study quality on the PEDro scale.

	Study	1*	2	3	4	5	6	7	8	9	10	11	Total
1	Mendes et al, 2013	✓	✓						✓		✓	✓	4
2	Ogalha et al, 2011	✓	✓		✓				✓		✓	✓	5
3	Mutimura et al, 2008	✓	✓		✓				✓		✓	✓	5
4	Hand et al, 2008	✓	✓		✓			✓			✓	✓	5
5	Filipas et al, 2006	✓	✓	✓	✓			✓	✓	✓	✓	✓	8
6	Dolan et al, 2006	✓	✓		✓				✓	✓	✓	✓	6
7	Rojas et al, 2003		✓		✓						✓	✓	4

1: eligibility criteria and source of participants; 2: random allocation; 3: concealed allocation; 4: baseline comparability; 5: blinded participants; 6: blinded therapists;7: blind assessors; 8: adequate follow-up; 9: intention-to-treat analysis; 10: between-group comparisons; 11: point estimates and variability.

*Item 1 does not contribute to the total score.

### Study Characteristics

The number of participants in the studies reviewed ranged from 3330 to 9726. Mean age of participants ranged from 37 to 46 years. Six studies included patients of both genders, one study included only women28, but there was an overall predominance of men. All studies analyzed in this review included outpatients diagnosed with HIV, most of them were in use of antiretroviral therapy. In five studies reviewed, all patients were receiving antirretrovial therapy. In the other two studies, about 85% of the participants were undergoing antiretroviral therapy.

Participants included adults infected with HIV at various stages of the disease with CD4 counts ranging from <100 to >500 cells/μl. In five studies reporting CD4 count, mean baseline value was 481.8 cells/μl. In these studies, there was no difference between participants in the CARE group compared to no exercise group and in the CARE group there was an increase of 12.6 cells/μl after the intervention. Sample size, outcomes and results of included studies are summarized in Table 3.

**Table 3:** Characteristics of the included studies.

		Outcome measures			
Study	Patients (N analysed, age, gender)	Aerobic capacity	Muscle Strength	HRQol	Key Findings
Mendes et al [24]	N = 80, 39.3 years, 63.8% female	Modified multi-stage fitness test	1-repetition maximum	NA	Strength and VO_2_ max improved in CARE group compared to No exercise (p = .05)
Ogalha et al [25]	N = 63, 43.2 years 54% male	Treadmill Stress Test	NA	SF-36	Peak VO_2_ and the domains of HRQoL (general health, mental health and vitality) improved in CARE group compared to No exercise (p = .05)
Mutimura et al [26]	N = 97, 37.7 years, 60% female	Shuttle test	NA	WHOQOL-BREF	Peak VO_2_, psychological, independence, social relationships and HIV+ HAART-specific domains of HRQoL improved in CARE group compared to No exercise (p = .05)
Hand et al [27]	N = 40, 41.8 years, 75% male	Graded Exercise Stress Test	NA	NA	Peak VO_2_ improved in CARE group compared to No exercise (p = .05)
Dolan et al [28]	N = 38, 41.5 years, 100% female	Treadmill Stress Test	1-repetition maximum	NA	Strength and VO_2_ max improved in CARE group compared to No exercise (p = .05)
Fillipas et al [29]	N = 35, 43.5 years, 100% male	Kasch Pulse Recovery test	NA	MOS-HIV	Cardiovascular fitness and the domains of HRQoL (overall health and cognitive function) improved in CARE group compared to No exercise (p = .05)
Rojas et al [30]	N = 33, 46.2 years, 70% male.	Graded Exercise Stress Test	NA	MOS-HIV	Peak VO_2_ and the domains of HRQoL (health status, emotional well-being, energy, physical strength and GQL) improved in CARE group compared to No exercise (p = .05)

HRQoL, Health-related quality-of-life; MOS-HIV, Medical outcomes study HIV health survey; GQL, Globat quality of life; NA, not assessed.

### Characteristics of Intervention Programs

Parameters used in the application of CARE have been reported in most studies, and all described the progressive nature of the exercise training. The characteristics of exercise intervention in included studies are provided in Table 4.

**Table 4:** Characteristics of the CARE Intervention in the Trials Included in the Review.

**Study**	Type exercise	Intensity/ duration (wk)	Volume	Frequency (x per Wk)	Time (min)	Length (wk)	Supervision
Mendes et al [24]	Aerobic exercise	50–70% HRmax	10 min warm up, 15–20 min exercise, 10 min cool down	3	40	24	Yes
	Resistance exercise	80% MR	3 sets, 8 reps	3	40	24	Yes
Ogalha et al [25]	Aerobic exercise	75% HRmax	15 min warm up, 60 min exercise, 15 min cool down	3	30	24	Yes
	Resistance exercise	NI	NI	3	30	24	Yes
Mutimura et al, 2008 [26]	Aerobic exercise	45% HRmax / 3, 60% HRmax / 6, 75% HRmax / 15	15 min warm up, 60 min exercise, 15 min cool down	3	90	24	Yes
	Resistance exercise	NI	NI	3	90	24	Yes
Hand et al, 2008 [27]	Aerobic exercise	50–70% HRmax	5 min warm up, 30 min exercise, 5 min cool down	2	40	6	NR
	Resistance exercise	12 MR	1 set -12 reps	2	20	6	NR
Dolan et al, 2006 [28]	Aerobic exercise	60% HRmax /, 275% HRmax / 14	5 min warm up, 20–30 min exercise	3	35	16	Yes
	Resistive exercise	60–70% MR/ 2, 80% MR/ 12	3–4 sets, 8–10 reps	3	85	16	yes
Filipas et al,2006 [29]	Aerobic exercise	60% HRmax / 3, 75% HRmax / 3	5 min warm up, 20 min exercise, 5 min cool down	2	30	6	Yes
	Resistive exercise	60% MR, 80% MR	3 sets, 10 reps	2	30	6	Yes
Rojas et al, 2003 [30]	Aerobic exercise	60–80% HRmax	10 min warm up, 25 min exercise, 10 min cool down	3	50	12	NR
	Resistive exercise	60–70% MR/ 4, 80% MR/ 12	2–3 sets, 8 reps	3	NI	12	NR

NR, Not reported; HRmax, maximum heart rate; MR, maximal repetition; Reps, repetitions.

### Peak VO_2_

Five studies (including 318 patients) assessed peak VO2 as outcome [24–28]. In the study of Mutimura et al [26] peak VO2 was improved from 4.7 ± 3.9 vs 0.5 ± 0.3 ml / kg per min in the intervention group compared to control (p <0.001), while the study of Hand et al [27], detected an improvement of 21% in peak VO2 estimated in the exercise group versus no improvement in the control group (p <0.001). Similarly, Dolan et al [28] observed an improvement (1.5 ± 0.8 vs -2.5 ± 1.6 mL/kgmin-1, p <0.001) in peak VO2 in the training group compared to control. However, Ogalha et al [25] observed a nonsignificant improvement (0.6 ± 0.9 vs -0.2 ± 0.7 mL/kgmin-1, p <0.001) in peak VO2 in the training group compared to control.

**Fig 2:**
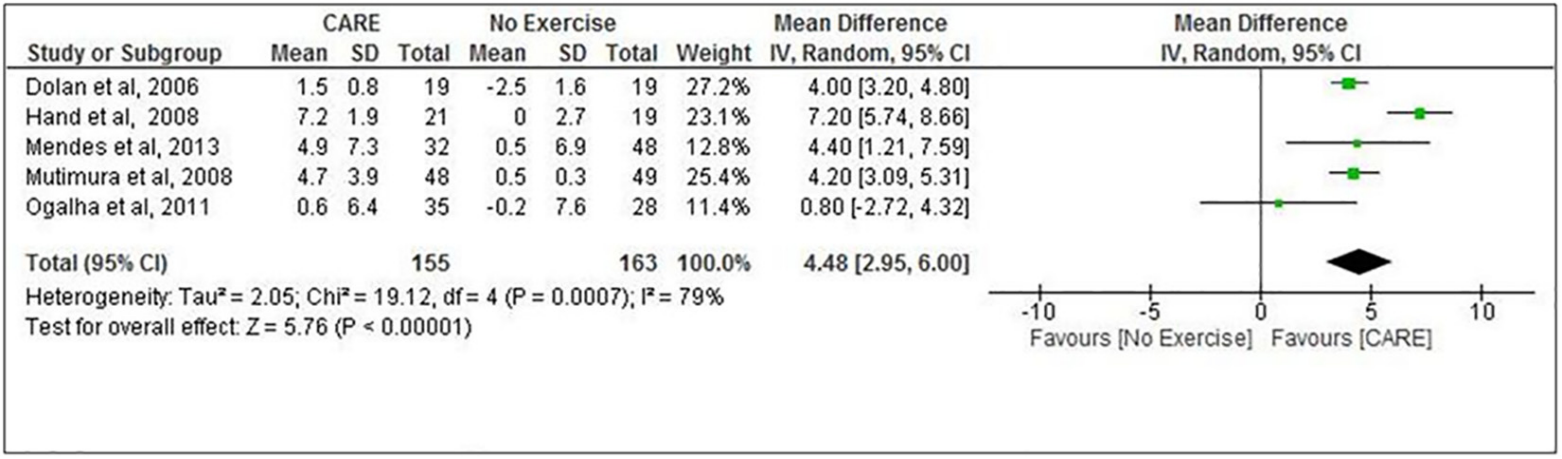
Combined aerobic and resistance exercise (CARE) versus No Exercise: Peak VO_2_. Review Manager (RevMan). Version 5.2 The Cochrane Collaboration, 2013.

The mean peak VO2 in the analyzed studies was 26.8 mL kg-1 min-1 at baseline, and it increased to 30.7 mL kg-1 min-1 at the end of the intervention. The meta-analyses showed (Fig 2) a significant improvement in peak VO2 of 4.48 mL kg-1 min-1 (95% CI: 2.95, 6.0, *N* = 318) for participants in the CARE group compared with no exercise group.

### Muscle Strength

**Fig 3:**
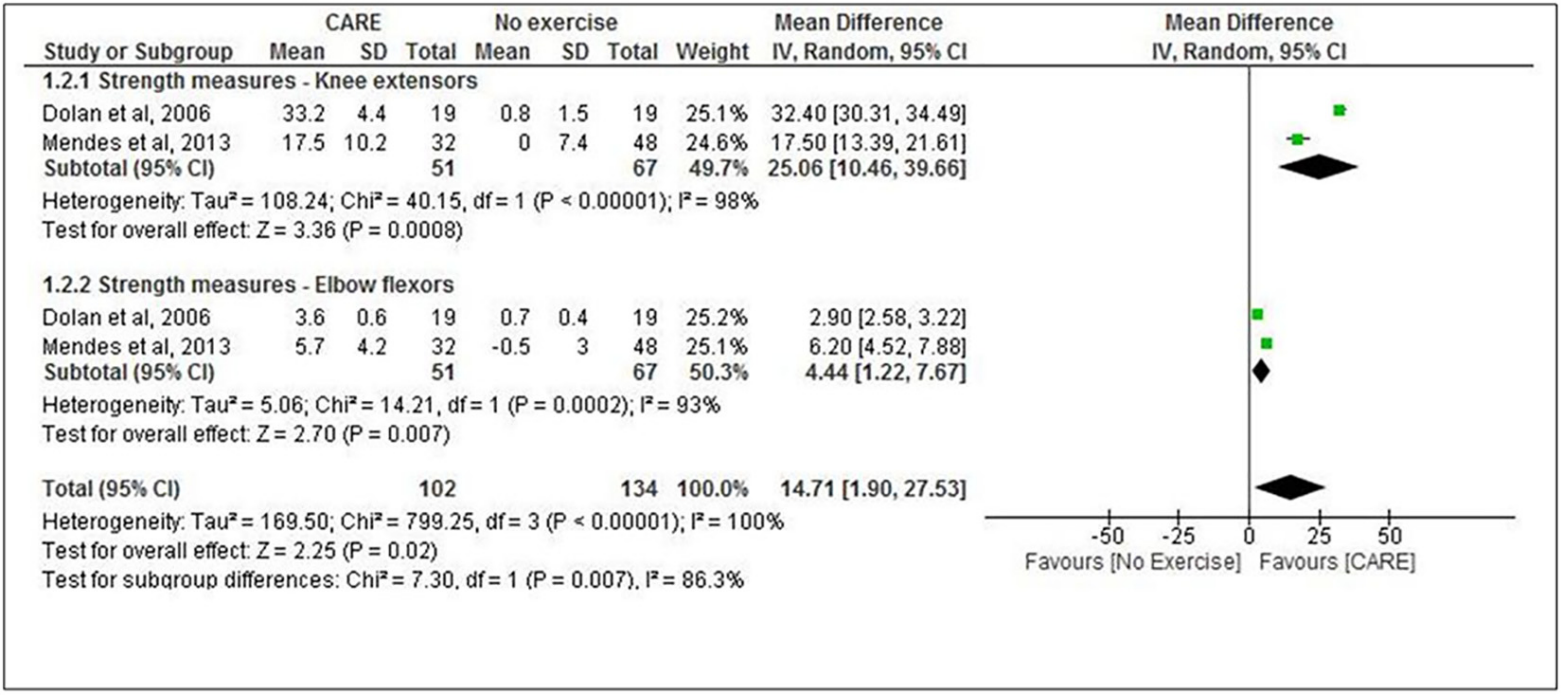
Combined aerobic and resistance exercise (CARE) versus No Exercise: Muscle Strength. Review Manager (RevMan). Version 5.2 The Cochrane Collaboration, 2013.

Two studies assessed muscle strength of the knee extensors and elbow flexors. [24,28] A total of 118 patients were included in these 2 studies. Mendes et al [24] and Dolan et al [28], showed a significant improvement in muscle strength of the knee extensors and elbow flexors. A significant improvement in muscle strength of the knee extensors of 25.06 Kg (95% CI: 10.46, 39.66 N = 118) and of 4.44 Kg (95% CI: 1.22, 7.67 N = 118) for Elbow flexors were found for participants in the CARE group compared with no exercise group. (Fig 3)

### Quality of Life

**Fig 4:**
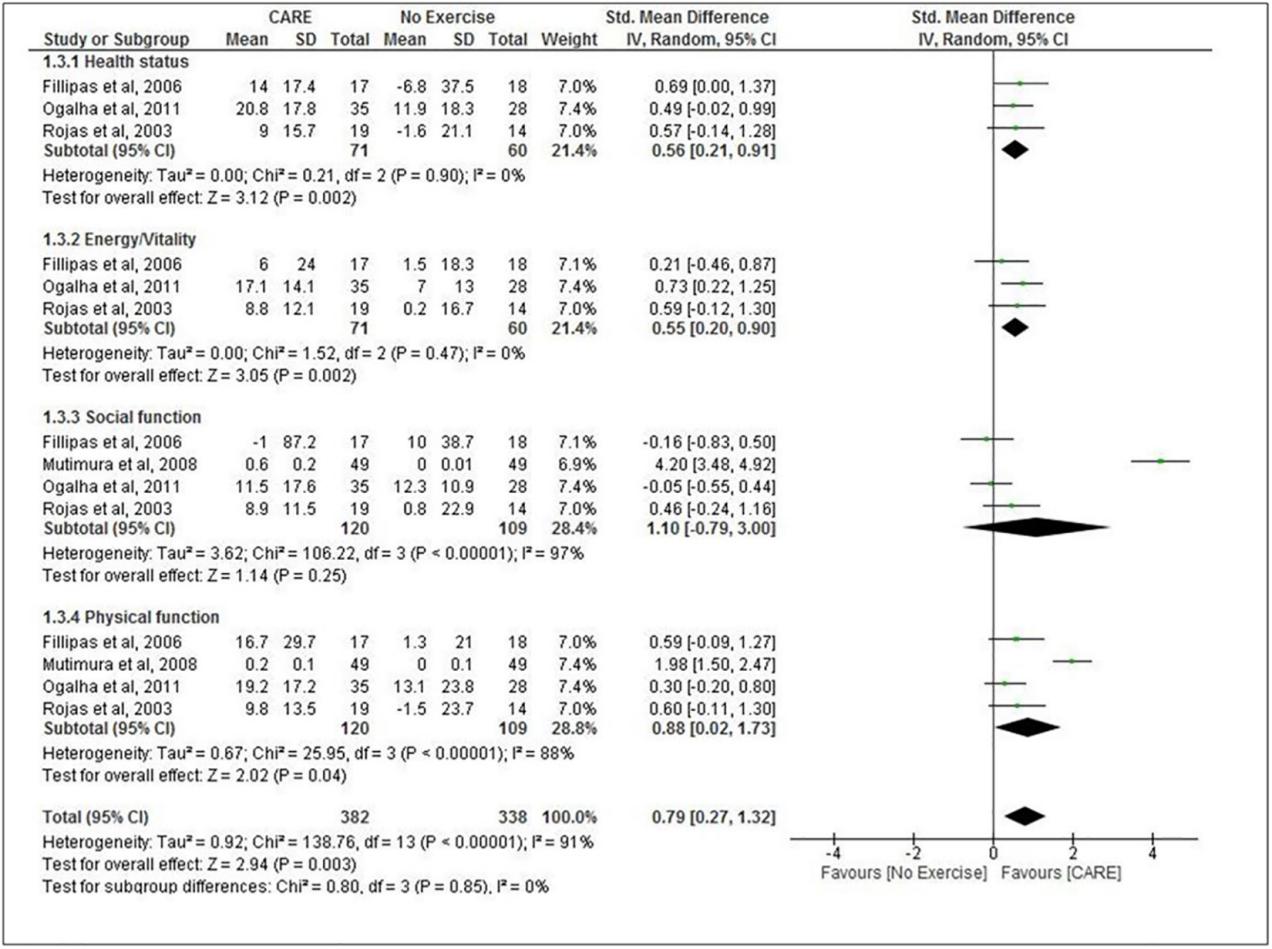
Combined aerobic and resistance exercise (CARE) versus No Exercise: HRQoL. Review Manager (RevMan). Version 5.2 The Cochrane Collaboration, 2013.

Four studies assessed quality of life [25,26,28,29]. A total of 229 patients were included in these 4 studies. In the study of Mutimura et al [26], quality of life showed a between-group difference in two dimensions (physical function and social function) while Ogalha et al [25], showed a between-group difference in only one out of the four dimensions in favor of CARE. Due to the difference between the instruments used in the assessment of quality of life, we performed a meta-analysis with standardized mean difference. Meta-analyses showed significant improvement in Health status, Energy/Vitality and Physical function domains of quality of life for participants in the CARE group compared with no exercise group (Fig 4). A non-significant improvement in social function domain was found for participants in the CARE group compared with no exercise group.

**Fig 5:**
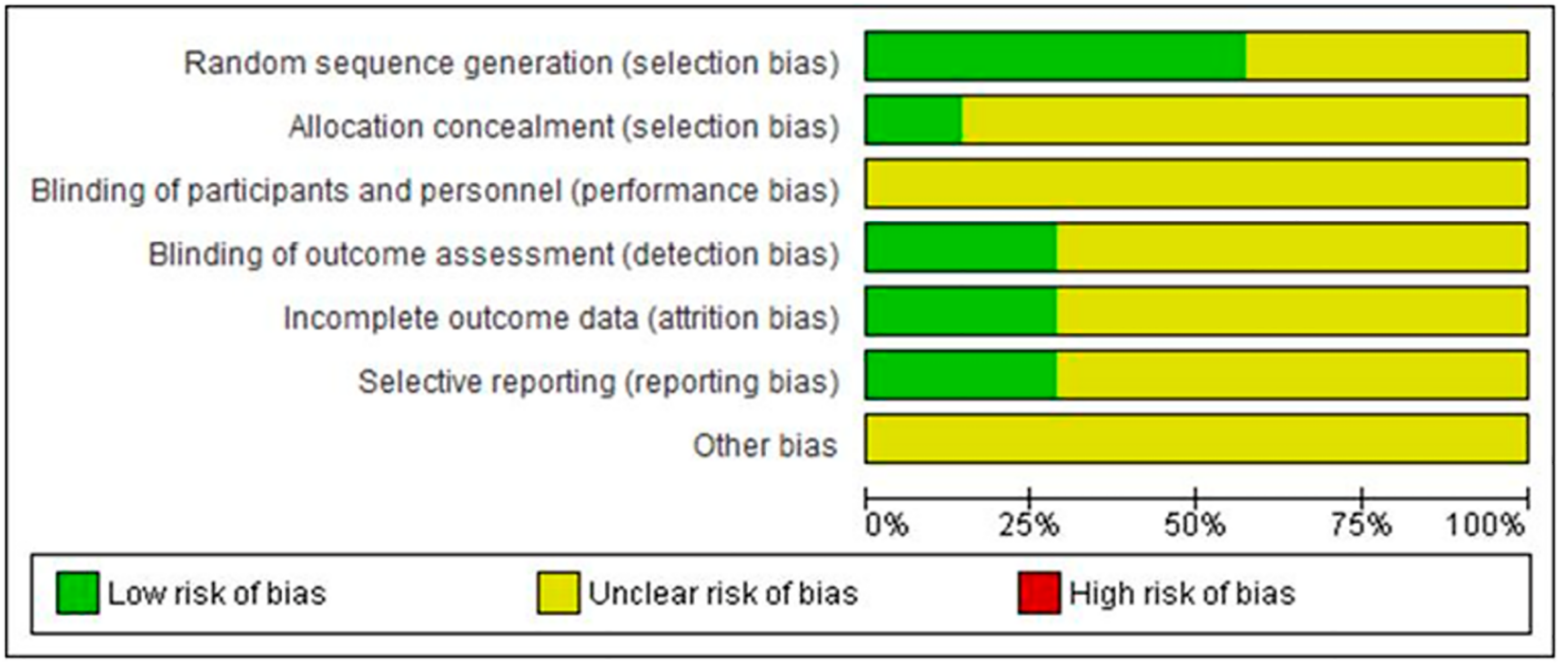
Risk of bias graph. Review Manager (RevMan). Version 5.2 The Cochrane Collaboration, 2013.

The analyzed studies did not give enough detail for potential risk of bias assessment. Details of the generation and concealment of the random allocation sequence were poorly reported. Only three studies presented objective evidence of the random allocation characteristics [27–29]. The studies presented objective evidence of balance in baseline characteristics. Two studies stated that the authors blinded the investigators involved in assessments [27,29]. The risk to selective reporting was uncertain and none of the studies described blinding of therapists. (Fig 5) summarizes the Risk of bias of included studies.

## Discussion

The main results of our meta-analyses indicate that CARE was efficient in increasing peak VO_2_, muscle strength and HRQOL in HIV-infected patients.

Exercise training is well established as an important nonpharmacological therapy in adult with chronic diseases, what is endorsed by the main guidelines around the world [31–33]. However, exercise training in HIV-infected patients is still poorly explored in scientific literature. Despite some available studies, we were unable to find any meta-analysis performed on CARE in HIV-infected patients, although recent reviews of progressive resistive exercise [9] and aerobic exercise [10] did include studies that used a combined approach. This systematic review with meta-analysis is relevant because it analyzes CARE as a potential co-adjuvant non-pharmacological treatment in this population. In addition, the eligibility of peak VO2 as outcome in this meta-analysis is important because it is the gold standard method to assess aerobic exercise capacity and is related to prognosis in patients with chronic conditions [34,35]. Moreover, quality of life is an essential component in a rehabilitation program.

The results of this review are in accordance with the findings of previous systematic reviews on progressive resistive exercise and aerobic exercise in adults living with HIV/AIDS [8–10]. However it is not possible to draw comparisons between the different types of exercise, due to lack of studies comparing the different types of exercise.

Reduced aerobic capacity may contribute to further deconditioning and activity limitations, placing HIV-infected patients at risk for poor health outcomes [36,37]. In our meta-analysis the mean of peak VO_2_ in the analyzed studies was 26.8 mL kg-1 min-1 at baseline, being 30.7 mL kg-1 min-1 at the end of the intervention. Specifically, the WMD in peak VO_2_ was 4.48 mL kg-1 min-1 favoring CARE, what represents an improvement of 16% in peak VO_2_. Considering peak VO_2_, it is known that improvements above 10% after a rehabilitation program are clinically meaningful in patient with chronic disease [35]. This improvement is similar to that found in programs of exercise for healthy people and can help patients to carry out their everyday activities [38].

Quality of life is a very important outcome in studies involving exercise training for HIV-infected patients. Antiretroviral therapy improves patients survival and quality of life by reducing the occurrence of HIV-related opportunistic infections [39,40]. Despite the patients in the studies included in the analysis were receiving antiretroviral therapy, our meta-analysis showed additional improvement in the domains physical function, health status and energy associated with the quality of life.

Raso et al [41] observed that poor muscle strength is observed in some HIV/AIDS patients, and is associated with lower anaerobic power and peak oxygen uptake. Studies in healthy people showed little impact of programs that combine aerobic and resistance exercise on muscle strength gain [42,43]. In this meta-analysis the mean of knee extensors muscle strength in the analyzed studies was 24.1 Kg at baseline, and increased to 49.2 Kg at the end of the intervention. For elbow flexors the baseline value was 9.7 Kg at baseline, increasing to 14.2 Kg at the end of the intervention. A minimal clinically important difference for muscle strength in HIV patients is not available. However, the gains were greater than 40% which likely represent a clinically meaningful strength gains. The detected improvement in muscle strength is important because it can be positively associated with the ability to perform daily living activities [44].

In accordance to the findings presented in this review, Ciccolo et al, suggests that exercise has the potential to be a beneficial treatment across the range of symptoms and adverse effects experienced by HIV-infected individuals. However, clinicians are currently advised to construct a general exercise programme to fit the individual needs, taking in account the stage of disease, symptoms and adverse effects experienced, besides functional ability [45].

CARE appears to be well tolerated by patients and combine the beneficial effects of both aerobic conditioning and skeletal muscle strength training. In our review, individual studies indicate that CARE appears to be safe for HIV-infected patients who are medically stable. The stability of immunological and virological measures during regular exercise can also be seen as evidence for the safety of this intervention [46,47].

Given the small amount of available studies and the significant heterogeneity found in the primary analyses due the variance in exercise protocols (variable intensities and different durations of the exercise programs), caution is warranted when interpreting our results.

The results of this systematic review should be cautiously interpreted due to a variety of reasons. It is possible that some participants in some studies were not excluded, due to co-infection with hepatitis C virus, as observed in the study of Perez et al [22]. Another limitation is that we did not compare CARE to aerobic or resistive exercise alone, so we still don't know if CARE is superior to aerobic or resistive exercise alone. Further investigation is required to investigate how to sustain positive effects of CARE in HIV-infected patient over time.

Further well controlled RCTs are required to reinforce the recommendation of exercise training as an important non-pharmacological treatment in HIV-infected patients. Additionally, it will be important to match exercise prescription to clinical/treatment characteristics of a patient subgroup or individual patient. It is important to determine the most appropriated methods (mode, intensity, frequency, duration, and timing) to achieve the best results regarding peak VO2 and quality of life.

## Conclusion

Taking in account the available studies, this meta-analysis showed that CARE is an efficient method to improve aerobic capacity, muscle strength and quality of life in HIV-infected patients. If no contraindications exist for individual patients, CARE should be considered as a component of care of HIV-infected individuals.

## Supporting Information

S1 Fig PRISMA Checklist. (DOC)

S1 File List of full-text excluded article. (DOCX)
