# Supplementary material for: Effects of Combined Aerobic and Resistance Exercise on Exercise Capacity, Muscle Strength and Quality of Life in HIV-Infected Patients: A Systematic Review and Meta-Analysis
Source: PLoS One. 2015 Sep 17;10(9):e0138066. doi: 10.1371/journal.pone.0138066 (PMC4574781; doi:10.1371/journal.pone.0138066)
Supplement: S1 File — (DOCX) [file pone.0138066.s002.docx]

 List of full-text excluded articles

Pérez-Moreno F, Cámara-Sánchez M, Tremblay JF, et al. Benefits of Exercise Training in Spanish Prison Inmates. Int J Sports Med 2007; 28:1-7

Reason for exclusion - the authors included HIV/HCV co-infected patient

[Driscoll SD](http://www.ncbi.nlm.nih.gov/pubmed?term=%22Driscoll%20SD%22%5BAuthor%5D) et al. Effects of exercise training and metformin on body composition and cardiovascular indices in HIV-infected patients. [AIDS.](http://www.ncbi.nlm.nih.gov/pubmed?term=6%20-%20Effects%20of%20exercise%20training%20and%20metformin%20on%20body%20composition%20and%20cardiovascular%20indices%20in%20HIV-infected%20patients) 2004;18(3):465-73.

Reason for exclusionThe authors linking exercise to other therapies.

[Grinspoon S](http://www.ncbi.nlm.nih.gov/pubmed?term=Grinspoon%20S%5BAuthor%5D&cauthor=true&cauthor_uid=10979879), [Corcoran C](http://www.ncbi.nlm.nih.gov/pubmed?term=Corcoran%20C%5BAuthor%5D&cauthor=true&cauthor_uid=10979879), [Parlman K](http://www.ncbi.nlm.nih.gov/pubmed?term=Parlman%20K%5BAuthor%5D&cauthor=true&cauthor_uid=10979879), [Costello M](http://www.ncbi.nlm.nih.gov/pubmed?term=Costello%20M%5BAuthor%5D&cauthor=true&cauthor_uid=10979879), [Rosenthal D](http://www.ncbi.nlm.nih.gov/pubmed?term=Rosenthal%20D%5BAuthor%5D&cauthor=true&cauthor_uid=10979879), [Anderson E](http://www.ncbi.nlm.nih.gov/pubmed?term=Anderson%20E%5BAuthor%5D&cauthor=true&cauthor_uid=10979879),et al. Effects of testosterone and progressive resistance training in eugonadal men with AIDS wasting. A randomized, controlled trial. [Ann Intern Med.](http://www.ncbi.nlm.nih.gov/pubmed/10979879) 2000;133(5):348-55.

Reason for exclusionThe authors linking exercise to other therapies.

Rigsby LW, Dishman RK, Jackson AW, Maclean GS, Raven PB. [Effects of exercise training on men seropositive for the human immunodeficiency virus-1.](http://www.ncbi.nlm.nih.gov/pubmed/1548998) Med Sci Sports Exerc. 1992;24(1):6-12.

Reason for exclusion -The authors investigated patients with and without HIV.
